# Association of Serum 25-Hydroxyvitamin D Levels With Acute Coronary Syndrome: A Case-Control Study

**DOI:** 10.7759/cureus.114101

**Published:** 2026-08-07

**Authors:** Krunal P Domki, Shilpa S Kuthe, Hitendra M Bhagwatkar, Vivek K Pande, Akshay S Shelar, Kartik M Khurana

**Affiliations:** 1 Department of General Medicine, NKP Salve Institute of Medical Sciences and Research Centre and Lata Mangeshkar Hospital, Nagpur, IND; 2 Department of Cardiology, NKP Salve Institute of Medical Sciences and Research Centre and Lata Mangeshkar Hospital, Nagpur, IND

**Keywords:** 25-hydroxy vitamin d, acute coronary syndrome, association, case control study, vitamin d

## Abstract

Background: Vitamin D deficiency has been proposed as a possible contributor to cardiovascular disease, but its relationship with acute coronary syndrome (ACS) remains uncertain.

Objective: This study assessed the association between serum 25-hydroxyvitamin D (25(OH)D) concentrations and ACS, including ST-elevation myocardial infarction (STEMI), non-ST-elevation myocardial infarction (NSTEMI), and unstable angina (UA), among patients presenting to a tertiary care center.

Methods: This hospital-based case-control study was done at the medical wards and intensive care unit (ICU) of NKP Salve Institute of Medical Sciences & Research Center and Lata Mangeshkar Hospital, Nagpur, India, for two years from 01/2021 to 12/2022, and included 140 participants, comprising 70 patients with ACS and 70 age- and sex-matched controls without ACS. Demographic characteristics, clinical history, cardiovascular risk factors, and laboratory findings were recorded. Serum 25(OH)D concentrations were measured using a chemiluminescent immunoassay and categorized as severe deficiency (<10 ng/mL), mild-to-moderate deficiency (10.0-19.9 ng/mL), insufficiency (20.0-29.9 ng/mL), or sufficiency (≥30 ng/mL). Multivariable logistic regression analysis was performed to determine the association between vitamin D status and ACS after adjustment for age and sex.

Results: Vitamin D deficiency and insufficiency were more frequent among patients with ACS than among controls. Participants with serum 25(OH)D concentrations below 30 ng/mL had significantly higher odds of ACS than those with sufficient vitamin D levels (adjusted odds ratio: 3.28; 95% confidence interval: 1.49-7.21; p = 0.003). Severe and mild-to-moderate vitamin D deficiency were also more common among ACS cases. However, serum vitamin D status was not significantly associated with the angiographic extent of coronary artery disease.

Conclusion: Lower serum 25(OH)D concentrations were significantly associated with ACS in this study population. However, the case-control design did not establish a causal or temporal relationship, and residual confounding could not be excluded. Larger prospective studies are required to clarify whether vitamin D deficiency independently contributes to ACS and whether its correction improves cardiovascular outcomes.

## Introduction

Acute coronary syndrome (ACS) is a term that refers to a group of clinical conditions that result from a sudden decrease in coronary blood flow, such as ST-elevation myocardial infarction (STEMI), non-ST-elevation myocardial infarction (NSTEMI), and unstable angina (UA) [1]. Although major progress has been made in cardiovascular prevention and treatment, ACS is still one of the most important causes of morbidity and mortality in the world. While well-established conventional cardiovascular risk factors include hypertension, diabetes mellitus, dyslipidemia, smoking, and other factors, novel biomarkers are gaining interest, as they could help more precisely estimate cardiovascular risk and provide further clues about disease pathogenesis [2]. Vitamin D is made mainly by the skin from exposure to sunlight, and it is also absorbed from the diet, but the amounts from the diet are relatively small in comparison with the amount of vitamin D made in the skin. Vitamin D, after synthesis, is hydroxylated in the liver to 25-hydroxyvitamin D (25(OH)D), which is the major circulating metabolite and best measure of vitamin D status. The biologically active form, calcitriol [3], is formed in a subsequent hydroxylation step in the kidneys. Although it's known to be important in calcium and bone metabolism, vitamin D has been linked with a number of cardiovascular processes. Experimental and clinical evidence indicate that vitamin D could play a role in endothelial function, in inflammatory responses, and in the regulation of the renin-angiotensin-aldosterone system (RAAS), thus affecting cardiovascular homeostasis [4].

Vitamin D receptors are found in vascular endothelial cells, vascular smooth muscle cells, and cardiomyocytes, which may contribute to the maintenance of vascular integrity and cardiac function. Vitamin D deficiency is linked to endothelial dysfunction, arterial stiffness, activation of the RAAS, and a pro-inflammatory and prothrombotic state. In addition, vitamin D regulates immune function by inhibiting pro-inflammatory cytokines such as interleukin-6 (IL-6) and tumor necrosis factor-alpha (TNF-α) and promoting anti-inflammatory cytokines such as IL-10, potentially leading to more stable plaques and decreased atherosclerotic progression [5]. Despite the abundance of sunshine in India, vitamin D deficiency is very common, ranging from about 50% to 90% of the population across various age groups and geographical regions. This is a common deficiency state that has resulted in significant interest in the role of this deficiency in the increasing load of cardiovascular disease in the Indian population [6].

There have been several studies exploring the association between vitamin D levels and cardiovascular disease, but the relationship between serum 25(OH)D levels and ACS has not yet been clear. A few studies have also reported that low levels of vitamin D are associated with a higher risk of coronary artery disease (CAD) and ACS [7]; however, others have not shown a significant association. Given the conflicting results reported and the existing high prevalence of both vitamin D deficiency and cardiovascular diseases in India, the present study was conducted to assess the correlation between serum 25(OH)D and ACS among the Indian tertiary care population.

## Materials and methods

This case-control study was done at the medical wards and intensive care unit (ICU) of NKP Salve Institute of Medical Sciences & Research Centre and Lata Mangeshkar Hospital, Nagpur, India, for two years from 01/2021 to 12/2022. The study was approved by the Institutional Ethics Committee (Approval No. NKPSIMS & RC and LMH/IEC/8/2021), and all participants gave written informed consent before enrollment. A total of 140 patients participated, including 70 ACS patients and 70 age- and sex-matched controls. Hospitalized adults (aged at least 18 years) presenting with a clinical picture and/or electrocardiogram (ECG) and cardiac biomarker findings consistent with a diagnosis of STEMI, NSTEMI, or UA were included. The control group consisted of age- and sex-matched adults admitted to the same medical wards of NKP Salve Institute of Medical Sciences and Research Centre and Lata Mangeshkar Hospital during the same study period as the ACS cases. Controls were admitted for noncardiovascular conditions, including uncomplicated gastrointestinal disorders, minor infections without sepsis, musculoskeletal complaints, or elective medical evaluation, and had no current or previous history of ACS or ischemic heart disease. Patients with heart failure, clinically significant arrhythmias, valvular heart disease, cardiomyopathy, peripheral arterial disease, cerebrovascular disease, or other established cardiovascular disorders were not included as controls because these conditions could independently influence serum vitamin D concentrations. Hospital-based controls were selected from the same medical wards and during the same recruitment period as the ACS cases to improve comparability with respect to geographic location, seasonal vitamin D exposure, healthcare access, and referral characteristics. Controls were admitted with noncardiovascular conditions that were not expected to substantially alter vitamin D metabolism. Patients with chronic kidney disease, chronic liver disease, thyroid or parathyroid disorders, malignancy, malabsorption, severe infection or sepsis, prolonged immobilization, or use of vitamin D supplements, glucocorticoids, anticonvulsants, or other drugs affecting vitamin D metabolism were excluded. The sample size was determined by the previously published study of vitamin D deficiency in 60% of patients with ACS and 35% of controls [8]. Based on a two-sided alpha of 0.05 and 80% power, the sample size of 64 subjects per group was the minimum necessary. For the possibility of incomplete data and as a means to enhance statistical precision, 70 subjects were recruited into each group for a total number of 140 subjects. Acute myocardial infarction was diagnosed according to the Fourth Universal Definition of Myocardial Infarction. Diagnosis required a rise and/or fall in cardiac troponin concentrations, with at least one value above the 99th-percentile upper reference limit, together with at least one feature of acute myocardial ischemia, including typical ischemic symptoms, new ischemic electrocardiographic changes, development of pathological Q waves, imaging evidence of new loss of viable myocardium or a new regional wall-motion abnormality, or identification of a coronary thrombus.

Data collection

Demographic data, clinical history, cardiovascular risk factors, and comorbidities such as hypertension and diabetes mellitus were recorded using a structured proforma. All participants had physical examination and anthropometric measures recorded. Both groups had laboratory analyses, such as complete blood count (CBC), fasting and postprandial blood glucose tests, glycosylated hemoglobin (HbA1c), blood pressures, ECG, and serum 25(OH)D concentrations. Along with ACS, patients were also evaluated for lipid profile, liver function tests, cardiac biomarker tests, two-dimensional echocardiography (2D echocardiography), and coronary angiography (CAG) to assess cardiac function and CAD. Using chemiluminescent immunoassay (CLIA), serum 25(OH)D level was determined. Vitamin D levels were defined as deficient (<20 ng/mL), insufficient (20.0-29.9 ng/mL), and sufficient (≥30 ng/mL).

Statistical analysis

Data were entered into MS Excel (Microsoft Corporation, Redmond, Washington, United States), and Stata version 10.1 (Released 2008; StataCorp LLC, College Station, TX, USA) software was used to analyze the data. Mean ± SD was used for continuous data, and frequencies and percentages for categorical data. The differences between groups were compared for continuous variables using the independent-samples t-test and for categorical variables using the Pearson chi-square test. The association of serum vitamin D status with ACS was assessed using multivariable logistic regression analysis, which was adjusted for potential confounding factors, and presented as odds ratios (ORs) with 95% confidence intervals (CIs). A two-sided p-value <0.05 was regarded as statistically significant.

## Results

The mean age was comparable between the case and control groups (59.0 ± 10.5 vs. 58.6 ± 10.2 years; p = 0.81). Similarly, waist circumference, systolic blood pressure, diastolic blood pressure, hemoglobin concentration, and platelet count did not differ significantly between the two groups (all p > 0.05). However, patients with ACS had significantly higher WBC counts (79.9 ± 23.4 × 10²/cumm vs. 71.6 ± 25.0 × 10²/cumm; t = 2.02, p = 0.04), fasting blood glucose levels (119.8 ± 32.7 mg/dL vs. 106.4 ± 26.0 mg/dL; t = 2.68, p = 0.008), and postprandial blood glucose levels (172.7 ± 48.4 mg/dL vs. 153.3 ± 47.4 mg/dL; t = 2.39, p = 0.01) than controls (Table 1).

**Table 1:** Baseline characteristics of study participants Values are presented as mean ± standard deviation. Comparisons between cases and controls were performed using the independent-samples t-test. A p-value of <0.05 was considered statistically significant

Variable	Cases (n = 70) mean ± SD	Controls (n = 70) mean ± SD	t	p-value
Age (years)	59.0 ± 10.5	58.6 ± 10.2	0.24	0.81
Waist circumference (cm)	80.6 ± 7.3	79.8 ± 6.8	0.67	0.50
Systolic blood pressure (mmHg)	129.1 ± 17.6	126.3 ± 13.7	1.06	0.29
Diastolic blood pressure (mmHg)	80.8 ± 11.7	79.4 ± 10.2	0.76	0.45
Hemoglobin (g/dL)	12.0 ± 1.2	12.1 ± 2.2	−0.28	0.78
Total leukocyte count (×10²/cumm)	79.9 ± 23.4	71.6 ± 25.0	2.02	0.04*
Platelet count (×10⁴/cumm)	23.0 ± 7.9	24.0 ± 9.9	−0.66	0.90
Fasting blood glucose (mg/dL)	119.8 ± 32.7	106.4 ± 26.0	2.68	0.008*
Postprandial blood glucose (mg/dL)	172.7 ± 48.4	153.3 ± 47.4	2.39	0.01*

The sex distribution was identical in both groups, with males comprising 74.3% of participants. Occupational distribution was comparable between cases and controls (χ² = 10.88; p = 0.054). Among the evaluated comorbidities, hypertension was significantly more prevalent in patients with ACS than in controls (48.6% vs. 24.3%; χ² = 8.91; p = 0.003). Smoking was also significantly more common among cases than controls (31.4% vs. 14.3%; χ² = 5.83; p = 0.016), whereas the prevalence of diabetes mellitus did not differ significantly between the two groups (31.4% vs. 20.0%; χ² = 2.39; p = 0.122) (Table 2).

**Table 2:** General characteristics of study participants Comparisons between cases and controls were performed using Pearson’s chi-square test. * indicates statistically significant p-value

Variable	Category	Cases (n = 70), n (%)	Controls (n = 70), n (%)	χ²	p-value
Gender	Male	52 (74.3)	52 (74.3)	0.000	1.000
	Female	18 (25.7)	18 (25.7)		
Occupation	Homemaker	9 (12.9)	8 (11.4)	10.88	0.054
	Self-employed	8 (11.4)	13 (18.6)		
	Semi-skilled	37 (52.9)	21 (30.0)		
	Skilled	9 (12.9)	12 (17.1)		
	Unemployed	5 (7.1)	7 (10.0)		
	Unskilled	2 (2.9)	9 (12.9)		
Comorbidities	Hypertension	34 (48.6)	17 (24.3)	8.91	0.003*
	Diabetes mellitus	22 (31.4)	14 (20.0)	2.39	0.122
	Smoking	22 (31.4)	10 (14.3)	5.83	0.016*

Chest pain was the predominant presenting symptom and was reported by all patients (100%). Other common presenting complaints included restlessness (44.3%), dyspnea on exertion (24.3%), and palpitations (15.7%). STEMI was the most frequent clinical presentation (84.3%), with anterior wall myocardial infarction representing the commonest subtype. NSTEMI and UA accounted for 8.6% and 7.1% of cases, respectively. On 2D echocardiography, hypokinesia was the most frequent finding (70.0%), whereas CAG most commonly demonstrated single-vessel coronary artery disease (61.4%) (Table 3).

**Table 3:** Clinical and cardiovascular findings in ACS cases (n = 70) ACS: acute coronary syndrome; STEMI: ST-elevation myocardial infarction; MI: myocardial infarction; NSTEMI: non-ST-elevation myocardial infarction; LVH: left ventricular hypertrophy Values are presented as frequency (percentage)

Parameter	Category	n (%)
Clinical presentation	Chest pain	70 (100.0)
	Restlessness	31 (44.3)
	Dyspnea on exertion	17 (24.3)
	Palpitations	11 (15.7)
ECG findings	STEMI	59 (84.3)
	Anterior wall MI	37 (52.9)
	Inferior wall MI	10 (14.3)
	Anteroseptal MI	5 (7.1)
	Inferoposterior MI	4 (5.7)
	Extensive anterior wall MI	3 (4.3)
	NSTEMI	6 (8.6)
	Unstable angina	5 (7.1)
2D echocardiography	Hypokinesia	49 (70.0)
	Concentric LVH	5 (7.1)
	Dilated cardiomyopathy	6 (8.5)
	Normal	10 (14.2)
Coronary angiography	Single-vessel disease	43 (61.4)
	Double-vessel disease	20 (28.6)
	Triple-vessel disease	2 (2.9)
	Normal coronaries	5 (7.1)

The distribution of serum vitamin D status and its association with ACS are shown in Table 4. Severe vitamin D deficiency (<10 ng/mL) was observed in 10.0% of cases compared with 4.3% of controls. Mild-to-moderate vitamin D deficiency (10.0-19.9 ng/mL) and vitamin D insufficiency (20.0-29.9 ng/mL) were also more frequent among patients with ACS. Conversely, vitamin D sufficiency (≥30 ng/mL) was more common in controls (41.4%) than in cases (18.6%). Overall, participants with serum vitamin D concentrations below 30 ng/mL had significantly greater odds of ACS (OR = 3.10; 95% CI: 1.36-7.28; p = 0.003) (Table 4).

**Table 4:** Association of vitamin D levels with ACS risk OR: odds ratio; CI: confidence interval; ACS: acute coronary syndrome Univariate logistic regression analysis was performed. p < 0.05 was considered statistically significant. * indicates a statistically significant p-value

Vitamin D level (ng/mL)	Cases (n = 70)	Controls (n = 70)	OR (95% CI)	p-value
<10 (severe deficiency)	7 (10.0)	3 (4.3)	5.25 (0.96-36.96)	0.022*
10-19.9 (mild–moderate deficiency)	28 (40.0)	18 (25.7)	3.47 (1.32-9.26)	0.005*
20-29.9 (insufficiency)	22 (31.4)	20 (28.6)	2.45 (0.92-6.61)	0.046*
≥30 (sufficiency)	13 (18.6)	29 (41.4)	0.32 (0.14-0.69)	0.003*

After adjustment for potential confounding variables, vitamin D deficiency or insufficiency (<30 ng/mL) remained significantly associated with ACS (adjusted OR = 3.28; 95% CI: 1.49-7.21; p = 0.003). Age and sex were not independently associated with ACS in the adjusted model (both p > 0.05) (Table 5).

**Table 5:** Multivariate logistic regression analysis of risk factors for ACS OR: odds ratio; CI: confidence interval; ACS: acute coronary syndrome Multivariable logistic regression analysis was performed after adjustment for potential confounding variables. * indicates a statistically significant p-value

Variable	Adjusted OR	95% CI	p-value
Vitamin D <30 ng/mL	3.28	1.49–7.21	0.003*
Age (years)	1.00	0.97–1.04	0.79
Gender (male)	0.91	0.41–2.01	0.81

## Discussion

The association between serum 25(OH)D concentrations and ACS in patients who presented to a tertiary care center was the subject of this hospital-based case-control study. The findings demonstrated that vitamin D deficiency and insufficiency were significantly more common among patients with ACS than among age- and sex-matched controls. After controlling for potential confounding variables, the association between serum vitamin D levels below 30 ng/mL and the risk of ACS remained statistically significant. The distributions of age and sex were broadly comparable across the two groups' initial demographics. Most participants were middle-aged men, reflecting the established epidemiology of CAD, which occurs more frequently in males and older adults. Among the conventional cardiovascular risk factors evaluated, hypertension was significantly more prevalent in patients with ACS, consistent with its established role in endothelial dysfunction, vascular remodeling, and atherosclerotic disease progression [9]. Smoking was also significantly more frequent among cases than controls, whereas the difference in diabetes mellitus prevalence was not statistically significant.

Laboratory findings further supported the presence of metabolic and inflammatory abnormalities among patients with ACS. Cases demonstrated significantly higher fasting and postprandial blood glucose levels than controls, findings that are consistent with the recognized contribution of hyperglycemia to endothelial injury, oxidative stress, and accelerated atherosclerosis [10]. In a similar vein, the inflammatory response associated with plaque instability and ACS is supported by significantly higher WBC counts in ACS patients [11]. The clinical profile of patients with ACS was consistent with previous reports [12]. Chest pain was the predominant presenting symptom, and STEMI represented the most common clinical presentation. On 2D echocardiography, most patients had hypokinesia, and CAG most often showed single-vessel coronary artery disease, which is consistent with the expected clinical spectrum of patients who present with acute coronary events. This study's main finding was that ACS was significantly linked to lower serum vitamin D levels. Severe deficiency, mild-to-moderate deficiency, and insufficiency were all more common among cases, whereas vitamin D sufficiency predominated among controls. Participants with serum vitamin D concentrations below 30 ng/mL had significantly greater odds of ACS, and the association remained significant after multivariable adjustment, supporting previous studies that have identified low vitamin D status as an independent marker of increased cardiovascular risk [13-15]. This association may be explained by a number of biological mechanisms. Cardiomyocytes, vascular smooth muscle cells, and vascular endothelial cells all express vitamin D receptors, suggesting that these cells play an important role in maintaining cardiovascular homeostasis [16]. Vitamin D deficiency has been linked to activation of the RAAS, endothelial dysfunction, and increased arterial stiffness, all of which may promote atherosclerotic disease [17]. In addition, vitamin D exerts immunomodulatory effects by suppressing pro-inflammatory cytokines and reducing vascular inflammation, processes that may influence plaque instability and thrombus formation [18]. These mechanisms provide biological support for the observed association between reduced vitamin D levels and acute coronary events [19]. However, due to the observational nature of the present study, it is appropriate to interpret these results as demonstrating an association rather than a causal connection. Although vitamin D deficiency has also been associated with insulin resistance and adverse metabolic profiles [20], these pathways were not directly evaluated in the present study.

Interestingly, serum vitamin D levels were not significantly associated with the angiographic extent of CAD. Although lower vitamin D concentrations were associated with the occurrence of ACS, they did not correlate with the number of diseased coronary vessels. Although larger prospective studies are required to confirm this observation, this finding suggests that vitamin D status may be more closely related to the occurrence of acute coronary events than to the anatomical extent of coronary artery involvement. Despite ample exposure to sunlight, this study found a high prevalence of vitamin D deficiency, which is consistent with previous reports from India. Factors including limited outdoor activity, increased skin pigmentation, urban lifestyles, and inadequate dietary intake have all been proposed as contributors to this paradox. The potential significance of identifying and treating vitamin D deficiency in populations at increased cardiovascular risk is further emphasized by these findings. 

This study has several strengths. The age- and sex-matched controls of the case-control design made it easier to compare the study groups. A standardized biochemical assay was used to measure serum vitamin D concentrations, and a multivariable logistic regression analysis was used to adjust for significant confounding variables, which strengthened the observed connection between vitamin D status and ACS. It's important to acknowledge a few limitations as well. This was a single-center study with a relatively modest sample size, which may limit the generalizability of the findings. The case-control design precludes establishing temporal or causal relationships between vitamin D deficiency and ACS. In addition, factors known to influence vitamin D status, including sunlight exposure, seasonal variation, dietary intake, physical activity, and socioeconomic status, were not evaluated and may have contributed to residual confounding.

## Conclusions

Vitamin D deficiency and insufficiency were more common among patients with ACS than among age- and sex-matched controls. However, the observational case-control design does not establish a causal or predictive relationship, and residual confounding by conventional cardiovascular risk factors cannot be excluded. These findings do not support routine vitamin D assessment for cardiovascular risk prediction but justify further prospective studies to clarify the temporal and clinical significance of this association.
